# Sepsis: Mr. Machado's *criteria*

**DOI:** 10.5935/0103-507X.20170037

**Published:** 2017

**Authors:** José Andrade Gomes

**Affiliations:** Unidade de Cuidados Intensivos, Hospital da Luz - Lisboa, Portugal.

I quite like Mr. João Machado.

I've known him for 2 or 3 years. An elderly man of various occupations, he had learned to survive through a curriculum made up of them all. Although I am usually unflappable, I believe that I would blush a little if he told me some of these chapters. Each of his hospitalizations has been due to acute decompensated heart failure that, I believe, is caused by hypertensive, ischemic, and perhaps (at least a little bit) alcoholic cardiopathy. He doesn't believe this; from atop his 1.8-m frame, which supports 83kg of good humor, he says he doesn't smoke much, doesn't drink too much, and doesn't neglect his high blood pressure all that much. He thinks that his heart is just worn out from suffering with many things he has seen during his 73 years. It's not impossible that he's right.

His last hospitalization was on July 5, 2016. He had basal crackles, abundant edema, an elevated creatinine level of 1.3mg/dL, and a peripheral oxygen saturation of 92% with 2L of oxygen by nasal cannula. Not so bad. Hemodynamically he was quite decent, with a blood pressure of 123/81mmHg and a heart rate of 72/minute in sinus rhythm; a daily whiff of beta blockers works wonders. The next day, Portugal played Wales in the semifinals of the European Football Championship, and we discussed whether Bruno Alves or Ricardo Carvalho should play instead of Pepe, who was injured; here, again, we disagreed.

With the Portuguese victory before 10 p.m. (a unique occurrence for the Portuguese team during this European tournament), Mr. Machado was euphoric. At 11:30 p.m., his temperature was 38.6ºC. Everyone blamed Ronaldo and Nani, who scored the goals (the *Klebsiella* remained blank). If I were in emergency care, I could believe the same thing. In any case, blood cultures and "inflammatory parameters" were taken.

On the 7^th^, Mr. Machado was still with good spirit and was waiting for me to admit that he was right when he insisted that Bruno Alves should play. His hemodynamic parameters continued to have similar values, and he was eupneic. He asked me why there had been that "pansy nonsense" (I know it's not nice to write it nowadays, but that's what he said) of collecting blood for analysis right at that moment. I told him that my colleague, in view of the fever, wanted to exclude an infection, which, in fact, was still likely because he had elevated white blood cells and C-reactive protein (13,400/mm^3^ and 6.3mg/dL, respectively). The blood cultures remained negative. He knew what I was talking about, as he had developed infections during previous hospital stays, and we had discussed the subject. He has a good memory.

- So, doctor, this is "*sepiss*"?

- Sepsis, Mr. Machado, it's pronounced "sepsis".

- Right, sepsis; it's like that other time, I have sepsis?

- Technically speaking, no, Mr. Machado, because the criteria for sepsis have changed since February 23 of this year.

- Ah, so it's not as dangerous as it used to be.

- Yes it is, Mr. Machado. Don't forget that for all intents and purposes, the disease is the same; what changed were the criteria for diagnosis.

- Oh, I see. Now it's easier to diagnose it.

- I can't say "yes" to that. Just to give you an example...in the old days, Mr. Machado, you met the criteria for sepsis, but now you don't; now your clinical condition is just called infection.

- Oh, doctor, just admit it: you doctors twist and half-change everything so that it's harder to get into the profession, don't you?

I smiled, told him two or three more things, and went to see other patients. The conversation with Mr. Machado had been more for affection, since he hadn't been assigned to me that day. I knew that my colleagues were thinking about performing one or two diagnostic tests and that it had been decided to treat him with antibiotics for a possible community-acquired respiratory tract infection.

On the 8^th^, he exhibited a similar air. His blood pressure and respiratory rate remained normal. He'd had two febrile episodes in the previous 24 hours, the leukocytosis persisted, and the C-reactive protein was still elevated, but his creatinine level was now 1.9mg/dL, and his urine output was 0.3 - 0.4mL/kg/hour.

- Doctor, here I am with my sepsis that isn't anymore!

Really, I love this man who always keeps such a positive attitude toward the disease.

- Look, Mr. Machado, I can tell you that under the "old" criteria, you would be classified as having severe sepsis, not just sepsis.

- And now I don't have it?

- As I told you yesterday, the disease hasn't changed; only the label. Therefore, the severity is the same.

- But what do you call it now, whatever it is I have today?

- You still have only the criteria for infection!

We drop Medicine and return to soccer. The previous evening, France beat Germany in the other semifinal match, and I thought that Portugal's chances of winning the Cup were minimal. Mr. Machado reassured me:

- If the disease is the same and the names change, maybe it's the same thing in soccer: the game is the same, but this time the title of champion goes to Portugal instead of going to France.

I thought to myself that that man would always believe, in every situation, in whatever suited him best.

July 9 was terrible for my patient. During the early hours, he was hypotensive and remained oliguric. He'd taken furosemide, with a slight diuretic effect, which also was not good for his blood pressure. In the morning, I found him hospitalized in the intermediate care unit. An arterial line and a central venous catheter were inserted, through which noradrenaline was perfused at 0.2 mcg/kg/minute to obtain an mean arterial pressure of approximately 73mmHg. Previously, two fluid challenges had been performed with no results. The arterial blood gas showed good oxygen partial pressure (106mmHg) with 2L/min oxygen by nasal mask. The levels of creatinine, white blood cells, and C-reactive protein were similar, but *Klebsiella* had been isolated from two blood cultures; fortunately, the strain was susceptible to amoxicillin clavulanate, but the bad news didn't end there: there had been an oversight, and no one had prescribed the antibiotic therapy decided on two days before! The only good news was that his lactate level was 6mg/dL (0.67mmol/L).

- Mr. Machado, I've come here to apologize.

- Why doctor?

- Remember when I told you we were going to start antibiotics? Well, by mistake, I didn't prescribe it, and we just now discovered that you weren't given antibiotics. It was only this morning that you started taking it.

- And you, doctor, you think it's too late, is that it?

- I really hope not, Mr. Machado; but if it had started two days ago, the infection probably wouldn't have worsened so much, and you'd be much better by now.

- So, with this worsening, now what do you call whatever I have?

- Now you have the criteria for sepsis, Mr. Machado.

- So, do you see, doctor? How I am now is how I used to be at the beginning of my hospital stay.

- I'm not following you, Mr. Machado.

- Doctor, you said that when I was hospitalized and I had a fever, that used to be sepsis; if it's sepsis that I have now, then I'm not worse, I'm the same.

- It's not quite like that. How you are today corresponds to what used to be called septic shock. And this is very serious and it's my fault.

- Oh, now there's no septic shock?

- No, there's still septic shock, but the criteria have changed, and you don't have the current criteria for septic shock, Mr. Machado.

- Doctor, doctor! I've seen a lot of change in my life, for better and for worse. But forgive me for saying... this is a change for nothing.

- Look, Mr. Machado, as long as you get well, I'm willing to agree with everything you say about Medicine today.

- I don't know much about Medicine, but I know about other things. Tomorrow I'll be getting better, and Portugal will be the European champion.

And you know that Mr. Machado was right!

José Andrade Gomes - Intensive Care Unit, Hospital da Luz - Lisboa, Portugal.

## Figures and Tables

**Table:** 

**Sepsis - 2**
Sepsis - SIRS + presumed infection

**Sepsis - 3**
Uncomplicated infection

qSOFA - 0

**Table:** 

**Sepsis-2**
Severe sepsis - sepsis + organ dysfunction

**Sepsis - 3**
Uncomplicated infection

qSOFA - 0

SOFA = 1

**Table:** 

**Sepsis - 2**
Septic shock - volume-refractory hypotension associated with sepsis

**Sepsis - 3**
Sepsis - volume-refractory hypotension associated with sepsis with normal lactate

SOFA = 6

